# Polymorphisms in Necroptosis Pathway Genes: Novel Prognostic Markers for Multiple Myeloma Treatment Outcomes

**DOI:** 10.3390/ijms26115237

**Published:** 2025-05-29

**Authors:** Marcin Sokołowski, Piotr Łacina, Katarzyna Bogunia-Kubik, Grzegorz Mazur, Aleksandra Butrym

**Affiliations:** 1 Dr Alfred Sokolowski Specialist Hospital in Walbrzych, 58-309 Wałbrzych, Poland; maxsokolonko@gmail.com; 2 Lower Silesian Oncology, Pulmonology and Hematology Center, 53-314 Wroclaw, Poland; 3 Laboratory of Clinical Immunogenetics and Pharmacogenetics, Hirszfeld Institute of Immunology and Experimental Therapy, Polish Academy of Sciences, 53-114 Wroclaw, Poland; katarzyna.bogunia-kubik@hirszfeld.pl; 4 Department of Emergency Medical Service, Wroclaw Medical University, 51-616 Wroclaw, Poland; grzegorzmaz@yahoo.com; 5 Department of Cancer Prevention and Therapy, Wroclaw Medical University, 50-556 Wroclaw, Poland; aleksandra.butrym@gmail.com; 6 Oncological Clinical Trials Support Center at Dr Alfred Sokolowski Specialist Hospital in Walbrzych, 58-309 Wałbrzych, Poland

**Keywords:** multiple myeloma, necroptosis, single-nucleotide polymorphism

## Abstract

Multiple myeloma is a neoplastic disease characterised by the proliferation of clonal, atypical plasma cells. In cancer cells, the balance between two paths of cell death, necroptosis and apoptosis, is disrupted. The aim of this study was to analyse the occurrence of polymorphisms in genes encoding key proteins for the necroptosis process, i.e., RIPK-1, RIPK-3 and MAPKAPK2. We investigated the potential relations between the occurrence of genetic variability and the clinical course of the disease. We analysed six single-nucleotide polymorphisms in a population of patients with multiple myeloma (*n* = 205) and healthy volunteers (*n* = 100): RIPK1 rs2272990, RIPK1 rs9391981, RIPK3 rs724165, RIPK3rs3212243, MAPKAPK2, rs45514798 and MAPKAPK2 rs4073250. We found that genotypes rs9391981 CG, rs724165 CG, rs3212243 GG, and rs4073250 AA were independent predictors of overall survival, while genotype MAPKAPK2 rs4073250 AA was an independent predictor of progression-free survival. MAPKAPK2 rs45514798 AA was associated with polyneuropathy after thalidomide therapy. In conclusion, some of the SNPs tested have potential prognostic value and could be used as marker of survival in patients with multiple myeloma.

## 1. Introduction

Multiple myeloma (MM) is a haematological malignancy characterised by proliferation of clonal, atypical plasma cells. It can lead to renal insufficiency, bone lesions, anaemia and hypercalcaemia. In the last 5 years, 230,000 cases of MM have been diagnosed, with a standardised ratio of 1.5 per 100,000 people [1]. The average age of diagnosis is 66–70; however, as many as 37% of patients were younger than 65 [2]. MM develops as a result of multistage transformation of differentiated plasma cells, resulting in constantly multiplying myeloma cells that produce and secrete abnormal immunoglobulins known as the M protein [3]. The oldest classification system of MM was created by Salmon and Durie in 1975 based on a host of clinical parameters [4]. A new, simpler classification known as the International Staging System (ISS) was presented in 2005, and its revised version is still commonly used in clinical practice [5,6,7]. MM treatment strategies constantly change due to the introduction of new therapies, although many older efficient groups of drugs are still in use. Proteasome inhibitors are commonly employed therapeutics which include bortezomib [8] and many second-generation therapeutics such as carfilzomib [9], delanzomib, oprozomib, and marizomib [10]. Immunomodulatory drugs (thalidomide, lenalidomide, and pomalidomide) are another class of potent anti-myeloma therapeutics used for MM treatment [11]. There are multiple clinical parameters that are of prognostic value in multiple myeloma, e.g., calcium blood level, haemoglobin, platelet count. Because of this, they are used by clinicians in routine practice for prognosis and staging of multiple myeloma [4,5,6,7].

Necroptosis is a programmed cell death pathway distinct from apoptosis. It shares some characteristics with the more unregulated necrosis, as both are caspase-independent and both result in a similar morphology of the dying cell. Similarly to apoptosis, the best-known activation pathway of necroptosis starts with tumour necrosis factor alpha (TNF-α) binding to its receptor TNFR1 on the cell surface [12]. This induces the formation of Complex I, which includes receptor-interacting serine/threonine kinase 1 (RIPK1) [13,14,15]. Complex I is stable due to polyubiquitination of RIPK1, and its deubiquitination leads to the formation of Complex II [16]. This new complex is formed around a scaffold made of RIPK1 and receptor-interacting serine/threonine kinase 3 (RIPK3), two proteins that are crucial in necroptosis [17]. At this point, the presence of activated caspase 8 decides whether the cell will follow the apoptotic or necroptotic pathway [18]. Necroptosis is initiated if caspase 8 is inhibited [19,20,21]. This leads to phosphorylation of RIPK1 and RIPK3, which is followed by their recruitment and activation of mixed lineage kinase domain-like pseudokinase (MLKL) [17,22,23,24,25,26,27,28,29]. RIPK1 and RIPK3, together with activated MLKL, form a necrosome, which then leads to cell death through permeabilisation of cell membrane, either due to uncontrolled Ca^2+^ influx or due to fragmentation of mitochondrial membrane by PGAM5 and DNM1L [30,31,32]. The consequence is a release of the alarmins–DAMP (damage-associated nuclear pattern) proteins characteristic of necrosis [33]. Formation of Complex II can be effectively inhibited by MAP kinase-activated protein kinase 2 (MAPKAPK2)-mediated phosphorylation of RIPK1. This event suppresses RIPK1 activation and blocks necroptosis, making MAPKAPK2-mediated phosphorylation a major survival checkpoint [34,35].

The aim of the present study was to analyse selected single-nucleotide polymorphisms (SNPs) in genes encoding proteins associated with necroptosis—RIPK1, RIPK3 and MAPKAPK2. Our previous studies showed that single-nucleotide polymorphisms (SNPs) located in genes crucial for myeloma cell survival may function as markers of survival and/or treatment outcome in MM patients [36,37,38]. We hypothesise that SNPs located in RIPK1, RIPK3 and MAPKAPK2 could be associated with multiple myeloma survival, treatment outcome and clinical parameters.

## 2. Results

### 2.1. Distribution of RIPK1, RIPK3, and MAPKAPK2 Genotypes in MM Patients and Healthy Controls

The distribution of the analysed polymorphisms was compared between MM patients and the control group. We observed no statistically significant differences between the two groups. The distribution of genotypes in both groups is presented in Table 1.

### 2.2. Associations with Diagnostic and Prognostic Parameters

**Figure 1:**
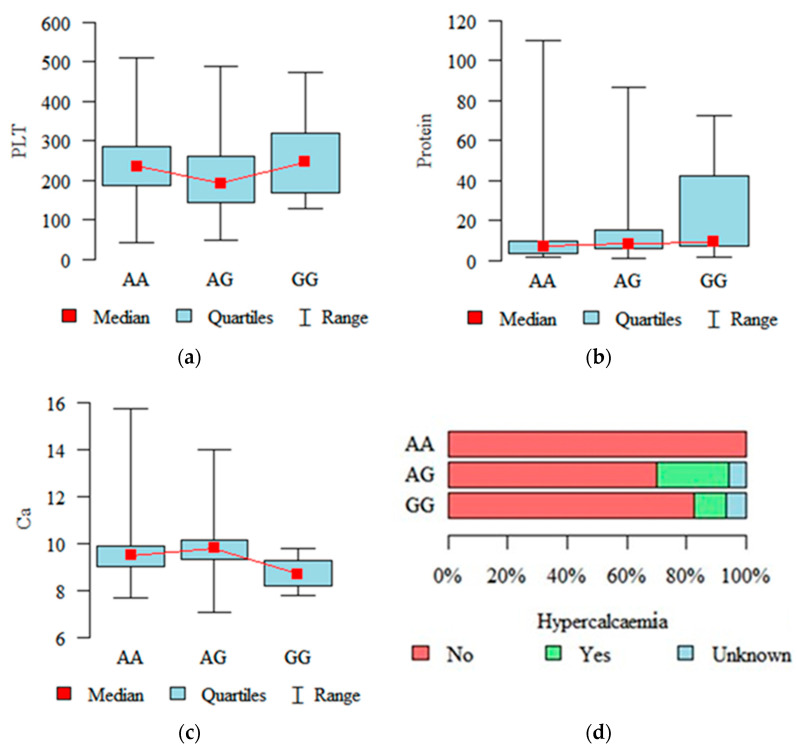
Associations between various clinical parameters in patients with multiple myeloma and RIPK3/MAPKAPK2 genotypes: (**a**) total serum protein level and RIPK3 rs724165 genotypes; (**b**) platelet count and RIPK3 rs724165 genotypes; (**c**) serum calcium level and RIPK3 rs3212243 genotypes; (**d**) hypercalcaemia and MAPKAPK2 rs4073250 genotypes.

Thrombocytopenia was observed in 18 patients (platelets < 100,103/µL), anaemia in 70 patients (haemoglobin < 10 G/L), leucocytosis in 17 patients (white blood cell count > 10,103/µL) and leukopenia in 44 patients (white blood cell count < 4 × 10^3^/µL). The average total protein concentration was 17.20 g/dL (median 8.40 dL), while the average concentration of beta-2-microglobulin was 5.74 g/dL (median 3.90 g/dL). The average creatinine serum level was 1.30 mg/dL (median 0.95 mg/dL). Patients with genotypes RIPK3 rs724165 GG and AA had a statistically higher platelet count than patients with genotype AG (*p* = 0.020, Figure 1a). However, RIPK3 rs724165 GG patients had a significantly higher level of total protein in serum compared to patients with genotype AA (*p* = 0.010, Figure 1b). Regarding the other SNPs, we observed that patients with genotype RIPK3 rs3212243 AG had statistically higher serum levels of calcium than patients with genotype GG (see Figure 1c). Moreover, we noted that hypercalcaemia was most common in the group of patients with variant MAPKAPK2 rs4073250 AG, and least common in patients with genotype AA (*p* = 0.039, Figure 1d).

### 2.3. Response to Treatment and Peripheral Polyneuropathy

**Figure 2:**
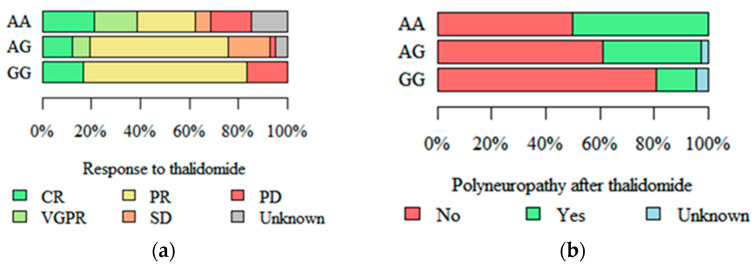
Response to treatment with thalidomide in patients with different RIPK3 rs3212243 genotypes (**a**), and peripheral polyneuropathy after thalidomide therapy, in patients with different MAPKAPK2 rs45514798 genotypes (**b**). Abbreviations: CR—complete remission, PR—partial remission, VGPR—very good partial remission, SD—stable disease, PD—progressive disease.

Differences in response to treatment with thalidomide were observed between patients with different RIPK3 rs3212243 genotypes. Both progressive disease (PD) and complete remission (CR) were significantly more common in patients with genotype GG as compared to other genotypes (*p* = 0.007, Figure 2a). Polyneuropathy as an adverse effect of thalidomide therapy was significantly more common among patients with MAPKAPK2 rs45514798 AA and less common in patients with genotype GG (*p* = 0.013, Figure 2b).

### 2.4. Stage in Durie–Salmon, ISS, and R-ISS Systems

**Figure 3:**
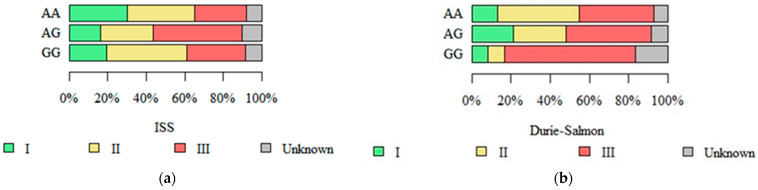
ISS stages and different RIPK3 rs724165 genotypes (**a**), and Durie–Salmon and different RIPK3 rs3212243 genotypes (**b**).

We observed an association between RIPK3 rs724165 genotypes and the stage of disease at diagnosis defined by the International Staging System (ISS). The stage of disease was the highest in patients with genotype AG, and the lowest in patients with genotype AA (*p* = 0.038, see Figure 3a). Furthermore, an association between RIPK3 rs3212243 and the stages defined by the Durie–Salmon staging system were also observed. Patients with genotype GG had a more advanced stage at diagnosis than patients with genotypes AA and AG (*p* = 0.035; see Figure 3b).

### 2.5. Survival Analysis

#### 2.5.1. Overall Survival

The median overall survival (OS) of all patients was 31 months. None of the analysed SNPs yielded any associations with OS in a univariate analysis. To account for the effect of potential confounding factors, we constructed a multivariate Cox proportional hazard model including various clinical parameters. The model includes RIPK1, RIPK3, and MAPKAPK2 SNPs as well as a number of clinical parameters. The analysis showed that the following genotypes are independent predictors of OS: RIPK1 rs9391981 CG, RIPK3 rs724165 CG, RIPK3 rs3212243 GG, MAP-KAPK2 rs4073250 AA (*p* < 0.05). Detailed results are presented in Table 2.

#### 2.5.2. Progression-Free Survival

**Figure 4:**
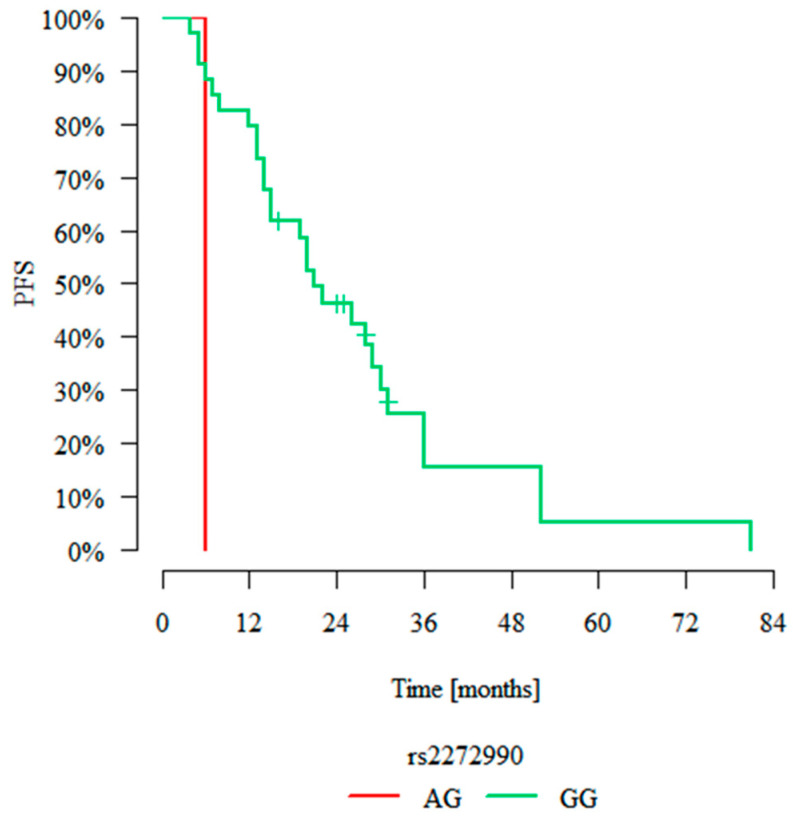
Progression-free survival (PFS) Kaplan–Meier curves for a subgroup of patients treated with lenalidomide carrying different RIPK1 rs2272990 genotypes.

The median progression-free survival (PFS) of all patients was 12 months. None of the analysed SNPs yielded any associations with PFS in a univariate analysis when analysing the whole group, although an analysis of a subgroup of patients treated with lenalidomide showed that genotype RIPK1 rs2272990 GG was associated with longer progression-free survival than genotype AG (*p* = 0.003, Figure 4). Similarly to OS, as described in the previous section, we analysed the effect of SNPs on PFS using a multivariate Cox proportional hazard model. It showed that only genotype MAPKAPK2 rs4073250 AA was an independent predictor of PFS (*p* = 0.025, HR = 6.919). Details of that analysis are shown in Table 3.

## 3. Discussion

The role of necroptosis in the pathogenesis of cancer, including multiple myeloma, has been proven many times before [39,40]. Ali and Mocarski showed that proteasome inhibitors cause a biochemical blockade of necroptosis in multiple myeloma, and mentioned that the interaction between myeloma cells and carfilzomib led to apoptosis [41]. This suggests that the mechanism of activity of proteasome inhibitors could be based on promotion of apoptosis and inhibition of necroptosis. Polymorphic variants of genes encoding proteins crucial for necroptosis may affect this process. Consequently, they could affect the clinical course of multiple myeloma, especially in patients treated with proteasome inhibitors. In the present study, we showed that SNPs located in genes encoding RIPK1, RIPK3 and MAPKAPK2 may be associated with the clinical parameters of multiple myeloma.

The first SNP analysed in the present study was rs9391981 C>G, an intron variant located in the gene coding for RIPK1 [42]. It has only been described once before, and it has been shown before that the occurrence of genotypes CG and GG increases the risk of multiple myeloma [43]. Although no such association was observed in our current study, we found that genotype CG was associated with worse overall survival in a multivariate analysis. The lack of association with risk may be due to a relatively small sample size, especially given the low minor allele frequency of this SNP. Another RIPK1 SNP investigated in this study, rs2272990 A>G, is a synonymous variant situated in a coding region. While synonymous variants do not affect the amino acid sequence of a protein, they can influence mRNA structure and stability, as well as protein folding [44]. Our survival analysis of patients treated with lenalidomide showed that patients with genotype AG had a worse prognosis than patients with genotype GG. In a similar study focusing on diffuse large B cell lymphoma (DLBCL), GG homozygosity was associated with a significantly shorter PFS [45]. However, this genotype was also a marker of longer OS in patients with colorectal cancer after the resection of primary lesion. Furthermore, patients with genotype AA and AG showed a significant reduction in disease-free survival (DFS) [46]. In a study of hepatocellular cancer, genotype GG correlated with a higher stage of TMN (tumour, nodes, metastases) and metastases in lymph nodes. GG homozygosity was significantly associated with severe hepatic ischemia-reperfusion injury and decreased survival after hepatectomy [47].

Although we observed an association between RIPK1 rs2272990 and progression-free survival, no statistically significant difference in overall survival was detected in our study. This could be related to a break in the natural balance between apoptosis and necroptosis in myeloma cells. This balance is dependent on a direct interaction between RIPK1 and RIPK3 by the means of trans- and autophosphorylation [39]. Longer PFS in the group of patients with genotype GG could be caused by apoptosis being favoured as a result of lenalidomide treatment. Lenalidomide stimulates T cells and increases the production of pro-apoptotic factors such as interleukin 2, interferon γ, and decreases the secretion of TNF-alfa. Cytotoxic T cells may activate apoptosis by ligation of FasL to FasR. Interleukin 2 could also stimulate lymphocytes and NK cells [48]. Furthermore, immunomodulatory agents may induce apoptosis by activating caspase 8, favouring the ligation of FasL and decreasing the NF-kappa B concentration. Therapy might also possibly block the stimulation effect of insulin-like growth factor 1 on NF-κB [49]. Interestingly, we did not observe any association between rs2272990 and response for any type of treatment.

The role of RIPK1 and necroptosis in carcinogenesis, as well as its effect on course of disease and prognosis, has been shown in many cancers [50]. In head and neck squamous cell carcinoma, RIPK1 downregulation increased the risk of metastasis through inhibition of NF-κB-pathway-dependent TLR3 receptor and by promoting apoptosis over necrosis [51]. The overexpression of RIPK1 was also detected in pancreatic adenocarcinoma cells and increased after chemotherapy. In vitro blockade of necrosome proteins promoted proliferation and led to an aggressive phenotype of pancreatic adenocarcinoma [51]. This suggests that RIPK1 may have a significant effect on cancer development. In the case of multiple myeloma, inhibition of necroptosis may lead to worse prognosis. The mechanism behind this process is unclear. As observed by Ali and Mocarski, proteasome initiates degradation of cIAP by secretion of SMAC/DIABLO from mitochondria [41]. This leads to inhibition of pro-survival processes and protects cells from necroptosis. The accumulation of cIAP2 may have a protective effect on cancer cells and may work against SMAC-dependent apoptosis [41]. After sensitising myeloma cells with cycloheximide, the degradation of cFLIP protein was observed [41]. In our study, we did not observe any association between RIPK1 SNPs (either rs2272990 or rs9391981) and response to proteasome inhibitor therapy. Further studies may be required to establish if there is any association between RIPK1 SNPs and response to proteasome inhibitors affecting necroptosis in multiple myeloma patients.

Our study also included rs724165 G>A, an intronic variant of RIPK3 [42]. We found it to be associated with the stage of disease using the ISS system, but not Durie–Salmon. ISS has been shown to have higher predictive value than Durie–Salmon staging in patients who did not undergo auto-HSCT. In our study, no association between rs724165 and response to auto-HSCT was found. Patients with genotype GG in locus RIPK3 rs724165 had a significantly higher level of total protein in serum than patients with the AA genotype. Furthermore, the level of platelets in the group with genotype GG and AA was significantly higher than for patients with genotype AG. Low platelet counts (<100,000/µL) may be a factor leading to a reduction in PFS and OS [52]. Other diagnostic and prognostic parameters analysed in our study (haemoglobin level, calcium serum level, creatinine serum level, kappa and lambda light chains in serum, ESR, ratio of plasma cells in bone marrow, and frequency of bone lesions) did not show any significant differences in patients with different rs724165 genotypes. To the best of our knowledge, rs724165 has only been described twice before. The first study showed an association between rs724165 and time to treatment, as well as OS [53]. The other study focused on non-Hodgkin lymphoma, and found that rs724165 is not a risk factor for that disease [54]. Due to its involvement in necroptosis, RIPK3 may play a role in cancer development [5]. Decreased expression of RIPK3 was connected with poor prognosis for women with breast cancer [55], and decreased OS and PFS in patients with colorectal cancer [46], as well as accelerated leukogenesis in patients with AML [56,57]. The other RIPK3 SNP included in our study, rs3212243, is located in the flanking region of the gene. We found that among patients with genotype AA, the frequency of CR and PD after thalidomide treatment was significantly higher than among patients with other genotypes. The explanation and significance of that phenomenon is unclear. Furthermore, patients with the GG genotype are characterised by a higher Durie–Salmon stage than patients with genotypes AG and AA. We also observed that the calcium serum level was significantly higher in carriers of genotype AG than in those with genotype GG.

We also analysed two SNPs located in the gene encoding MAPKAPK2–rs45514798 and rs4073250. The former is an intronic variant, while the latter is a synonymous SNP located in the coding regions of MAPKAPK2. Little is known about these two SNPs. In the case of rs45514798, is has been analysed in relation to HDL serum level in a Uyghur population in Xinjiang, China. In a cohort consisting of men under 50, rs45514798 genotypes were found to be associated with serum level of HDL [58]. However, no association with either serum level of TNF-alpha or LDL was observed [59]. Our study is the first to analyse rs4073250. We proved that rs4073250 was associated with the calcium level in serum, with a higher calcium level being observed in AG heterozygotes. Regarding rs45514798, we showed that polyneuropathy after thalidomide therapy was most frequent in AA homozygotes. It has been shown previously that necroptosis may have a role in neuron injury and Parkinson’s disease [60,61,62]. Necroptosis may also have a role in axial degeneration of the central and peripheral nervous systems. This could partially explain the pathophysiology of neurodegenerative disease and adverse events after chemotherapy [63]. The exact mechanism of thalidomide-induced neurotoxicity is not clear. The antiangiogenic effect of thalidomide could lead to ischaemia and hypoxia of nerves [64]. Another possible mechanism is decreased expression of TNF-alfa and inhibition of NF-κB [55]. Oxidative damage of DNA is also postulated [17]. To the best of our knowledge, there are no studies describing any association between MAPKAPK2 and polyneuropathy in any disease. One study focusing on motor neuron disease showed a crucial role of SHSP1 (small heat shock protein 1), which is phosphorylated by MAPKAPK2 [20]. Mutations in the gene coding for SHSP1 (R127W and R135F) tend to form small oligomers, which increase phosphorylation of MAPKAPK2. That process could play a role in neuropathy in Charcot–Marie–Tooth disease [20].

A multivariate Cox proportional hazard model analysis proved that RIPK1 rs9391981 CG, RIPK3 rs724165 GG, RIPK3 rs3212243 GG, and MAPKAPK2 rs4073250 AA are independent prognostic factors of shorter overall survival. In the case of rs724165, this seems to be in line with other results showing that rs724165 GG is associated with high total protein at diagnosis. Likewise, it is in line with our findings that genotype rs3212243 GG is more common in patients with more advanced disease. A separate multivariate analysis also proved the significance of MAPKAPK2 rs4073250 AA as an independent prognostic factor of shorter PFS in patients with MM.

It should be acknowledged that the treatment regimens used in the studied cohort partly reflect earlier therapeutic standards, which may represent a limitation of this study in the context of current multiple myeloma treatment paradigms. However, it is important to emphasise that the fundamental mechanisms of action of the main drug classes—particularly immunomodulatory drugs (thalidomide, lenalidomide) and proteasome inhibitors (bortezomib)—remain unchanged and still form the foundation of contemporary therapeutic protocols. Moreover, the molecular mechanisms influencing necroptosis processes that we investigated are universal and independent of specific treatment regimens. The associations we observed between polymorphisms in RIPK1, RIPK3, and MAPKAPK2 genes and patient survival and treatment response reflect deeper biological relationships between genetic variants of necroptosis pathways and the pathophysiology of multiple myeloma. The value of the presented findings lies in their potential as prognostic biomarkers that could aid in patient risk stratification regardless of the treatment schemes employed. Furthermore, the identified polymorphisms may represent promising targets for future research on novel targeted therapies that could modulate necroptosis pathways to enhance treatment efficacy. In this context, our discoveries maintain significant scientific and clinical value, opening new perspectives for understanding the biological basis of multiple myeloma and personalising therapeutic approaches.

In summary, this is the first study to analyse the effect of SNPs in genes associated with necroptosis in a group of patients with multiple myeloma. Some of the SNPs have a potentially prognostic value and could be useful in clinical practice. Genotypes rs9391981 CG (RIPK1), rs724165 GG (RIPK3), rs3212243 GG (RIPK3) and rs4073250 AA (MAPKAPK2) were found to be independent prognostic factors connected with a reduction in overall survival in patients with multiple myeloma, while genotype rs4073250 AA (MAPKAPK2) also led to reduction in progression-free survival. We also proved that patients treated with lenalidomide who carried genotype rs2272990 GG (RIPK1) had a longer progression-free survival than those with AG heterozygotes. Polyneuropathy as an adverse effect of therapy with thalidomide was significantly more common in patients with genotype rs45514798 AA (MAPKAPK2) and least common in patients with genotype GG. These results suggest that SNPs in RIPK1, RIPK3 and MAPKAPK2, genes associated with necroptosis, may have an effect on multiple myeloma development and treatment. However, further studies on larger groups of patients may be required to confirm these results.

## 4. Materials and Methods

### 4.1. General Characteristic of Population

The clinical group was composed of 205 patients with multiple myeloma (102 women and 103 men) from the Alfred Sokołowski Specialist Hospital in Wałbrzych, Poland, and from the Clinical University Hospital in Wrocław, Poland. The inclusion criteria were age over 18 and a multiple myeloma diagnosis. Written informed consent was obtained from all participants. This project received a positive opinion of the Bioethical Committee of Medical University in Wrocław. This study was conducted in accordance with the ethical standards of the Declaration of Helsinki from 2013. The median age in the group of patients was 65 years (the youngest was 37 and the oldest was 86). Detailed information about the patients is included in Table 4.

Renal insufficiency was present in 55 patients, 32 patients had hypercalcaemia, and 99 had osteolytic lesions in bones. The secretion of IgG M protein was detected in 118 patients, IgA–in 45, IgD–1, IgM–1, and 1 patient secreted two classes of M protein, IgG and IgM. Non-secretory multiple myeloma was diagnosed in 23 cases and 1 participant had plasma cell leukaemia. The control group was composed of 100 healthy volunteers (58 women and 48 men), aged 27 to 76 years (median 59 years).

The treatment was as follows: 100 patients were treated with bortezomib, 46 with lenalidomide, and 124 with thalidomide in various lines of therapy. The participants received, on average, 2 lines of therapy (1 to 4). Thalidomide was given to 91 patients, bortezomib to 91 patients and lenalidomide to 62 patients. Megachemotherapy before autologous haematopoietic stem cell transplantation (auto-HSCT) was applied to 58 patients. Polyneuropathy was observed in 33 patients as a result of previous therapy (16 after thalidomide, 9 after bortezomib, 3 after lenalidomide—probably not associated with therapy—and 4 cases had unclear aetiology and could have been caused by diabetes mellitus or many lines of therapy).

### 4.2. Genotyping

DNA was isolated from samples of peripheral blood taken on EDTA during routine laboratory tests using Maxwell 16 Blood DNA Purification Kit (Promega Corp., Madison, WI, USA) or silica membranes (Qiagen, Hilden, Germany), following the manufacturers’ recommendations. Specific RIPK1, RIPK3, and MAPKAPK2 SNPs were selected based on previous studies [43,45,47,54,58] and the results from the SNP Function Prediction tool [42]. To detect genotypes, LightSNiP assays (TIB MOLBI-OL, Berlin, Germany) and the real-time PCR (Polimerase Chain Reaction) method with melting curve analysis were used. Reactions were performed in a LightCycler 480 II (Roche Diagnostics GmbH, Rotkreuz, Switzerland) device, according to the recommendations of the manufacturer.

### 4.3. Statistical Analysis

A comparison of the qualitative variables between groups as performed by the chi-squared test (with Yates correction) or Fisher’s exact test. Comparisons of quantitative variables between two groups were performed using the Mann–Whitney U test. Comparisons of three and more groups were performed by Kruskal–Wallis test and post hoc Dunn analysis if the difference was statistically significant. The multivariate analysis was performed by logistic regression. The results are presented as an odds ratio with a 95% confidence interval. A comparison of survival was performed by the long-rank test. Multivariate survival analysis was calculated using the Cox proportional hazard model. The results are presented as hazard ratios with a 95% confidence interval. *p*-values < 0.05 were considered statistically significant.

## Figures and Tables

**Table 1:** Distribution of genotypes in MM patients.

	MM Patients	Control Group
**RIPK1 rs2272990**		
GG	188	94
AG	17	6
AA	0	0
**RIPK1 rs9391981**		
GG	189	92
CG	16	8
CC	0	0
**RIPK3 rs724165**		
AG	106	52
AA	63	34
GG	36	13
**RIPK3 rs3212243**		
AA	122	54
AG	71	44
GG	12	2
**MAPKAPK2 rs4073250**		
GG	130	58
AG	67	31
AA	4	8
**MAPKAPK2 rs45514798**		
GG	139	73
AG	62	24
AA	4	3

**Table 2:** Multivariate Cox proportional hazard model analysis of overall survival. Statistically significant *p*-values are indicated in **bold**.

Feature		HR	95% CI		*p*-Value
Sex	female	1	ref.		
	male	0.423	0.105	1.703	0.226
Age	[years]	1.246	1.115	1.392	**<0.001**
Durie–Salmon	I	1	ref.		
	II	2.077	0.228	18.927	0.517
	III	23.261	2.007	269.590	**0.012**
ISS	I	1	ref.		
	II	1.656	0.201	13.608	0.639
	III	0.102	0.004	2.518	0.163
Type of light chain	Lambda	1	ref.		
	Kappa	12.533	2.016	77.919	**0.007**
Type of heavy chain	A	1	ref.		
	G	12.533	0.058	1.202	0.085
Haemoglobin		0.920	0.597	1.417	0.706
Hypercalcaemia	No	1	ref.		
	Yes	0.185	0.015	2.240	0.185
Renal insufficiency	No	1	ref.		
	Yes	1.154	0.225	5.933	0.864
Osteolysis	No	1	ref.		
	Yes	4.186	0.851	20.591	0.078
RIPK1 rs2272990	GG	1	ref.		
	AG	*No deaths—impossible to calculate*			
RIPK1 rs9391981	GG	1	ref.		
	CG	71.614	2.333	2198.070	**0.014**
RIPK3 rs724165	AA	1	ref.		
	AG	0.567	0.089	3.600	0.548
	GG	<0.001	<0.001	0.144	**0.009**
RIPK3 rs3212243	AA	1	ref.		
	AG	5.199	0.679	39.795	0.112
	GG	1389.587	3.493	552,848.394	**0.018**
MAPKAPK2 rs4073250	GG	1	ref.		
	AG	1.029	0.214	4.941	0.971
	AA	30.671	1.356	693.526	**0.031**
MAPKAPK2 rs45514798	GG	1	ref.		
	AG	2.233	0.488	10.230	0.301
	AA	*No deaths—impossible to calculate*			

**Table 3:** Multivariate Cox proportional hazard model analysis of progression-free survival. Statistically significant *p*-values are indicated in **bold**.

Feature		HR	95% CI		*p*-Value
Sex	female	1	ref.		
	male	1.009	0.531	1.918	0.978
Age	[years]	1.019	0.985	1.055	0.275
Durie–Salmon	I	1	ref.		
	II	0.648	0.244	1.720	0.384
	III	0.753	0.266	2.125	0.591
ISS	I	1	ref.		
	II	1.666	0.636	4.366	0.299
	III	1.139	0.353	3.677	0.828
Type of light chain	Lambda	1	ref.		
	Kappa	1.639	0.797	3.370	0.179
Type of heavy chain	A	1	ref.		
	G	1.050	0.515	2.143	0.893
Haemoglobin		0.897	0.738	1.092	0.279
Hypercalcaemia	No	1	ref.		
	Yes	1.373	0.476	3.956	0.557
Renal insufficiency	No	1	ref.		
	Yes	1.226	0.655	2.293	0.524
Osteolysis	No	1	ref.		
	Yes	0.885	0.467	1.679	0.709
Response to the first line of treatment	CR	1	ref.		
	VGPR	0.872	0.281	2.706	0.813
	PR	1.112	0.502	2.463	0.793
	SD	1.046	0.406	2.700	0.925
	PD	0.357	0.085	1.503	0.160
RIPK1 rs2272990	GG	1	ref.		
	AG	0.676	0.059	7.732	0.753
RIPK1 rs9391981	GG	1	ref.		
	CG	1.207	0.1	14.611	0.882
RIPK3 rs724165	AA	1	ref.		
	AG	1.513	0.697	3.285	0.295
	GG	0.645	0.218	1.909	0.428
RIPK3 rs3212243	AA	1	ref.		
	AG	0.786	0.382	1.619	0.514
	GG	2.265	0.453	11.316	0.319
MAPKAPK2 rs4073250	GG	1	ref.		
	AG	1.125	0.548	2.308	0.749
	AA	6.919	1.277	37.473	**0.025**
MAPKAPK2 rs45514798	GG	1	ref.		
	AG	1.884	0.945	3.757	0.072
	AA	3.060	0.264	35.46	0.371

**Table 4:** Patients’ characteristics.

	N = 205
Sex	
female	102 (49.8%)
male	103 (50.2%)
Age	
median	65 years
range	37–88 years
Immunoglobulin secretion	
IgG	118 (57.6%)
IgA	45 (22.0%)
IgM	1 (0.5%)
IgD	1 (0.5%)
IgM and IgG	1 (0.5%)
Light chain	15 (7.3%)
Plasma cell leukemia	1 (0.5%)
Non-secretory	23 (11.2%)
ISS stage	
I	44 (21.5%)
II	67 (32.7%)
III	80 (39.0%)
unknown	14 (6.8%)
Durie–Salmon stage	
I	33 (16.1%)
II	72 (35.1%)
III	88 (42.9%)
unknown	12 (5.9%)
Therapy	
thalidomide	91 (44.4%)
bortezomib	91 (44.4%)
lenalidomide	62 (30.2%)
auto-HSCT	58 (28.3%)
Other parameters	
renal insufficiency	55 (26.8%)
hypercalcaemia	32 (15.6%)
osteolytic lesions	99 (48.3%)

## Data Availability

The data presented in this study are available on request from the corresponding author.

## Abbreviations

The following abbreviations are used in this manuscript:

MM	Multiple myeloma
ISS	International Staging System
TNF-α	Tumour necrosis factor alpha
RIPK1	Receptor-interacting serine/threonine kinase 1
RIPK3	Receptor-interacting serine/threonine kinase 3
MLKL	Mixed lineage kinase domain-like pseudokinase
DAMP	Damage-associated nuclear pattern
MAPKAPK2	MAP kinase-activated protein kinase 2
SNP	Single-nucleotide polymorphism
CR	Complete remission
PR	Partial remission
VGPR	Very good partial remission
SD	Stable disease
PD	Progressive disease
OS	Overall survival
PFS	Progression-free survival
DLBCL	Diffuse large B cell lymphoma
DFS	Disease-free survival
TMN	Tumour, nodes, metastases
SHSP1	Small heat shock protein 1
auto-HSCT	Autologous haematopoietic stem cell transplantation
